# Pneumonitis Incidence in Patients With Metastatic Non-small Cell Lung Cancer on Immunotherapy: A Systematic Review and Meta-Analysis

**DOI:** 10.7759/cureus.63615

**Published:** 2024-07-01

**Authors:** Sakditad Saowapa, Natchaya Polpichai, Pharit Siladech, Chalothorn Wannaphut, Manasawee Tanariyakul, Phuuwadith Wattanachayakul, Pakin Lalitnithi

**Affiliations:** 1 Internal Medicine, Texas Tech University Health Sciences Center, Lubbock, USA; 2 Internal Medicine, Weiss Memorial Hospital, Chicago, USA; 3 Internal Medicine, Ramathibodi Hospital, Chiang Mai, THA; 4 Internal Medicine, John A. Burns School of Medicine, University of Hawaii, Honolulu, USA; 5 Internal Medicine, Einstein Medical Center Philadelphia, Philadelphia, USA; 6 Internal Medicine, St. Elizabeth's Medical Center, Boston, USA

**Keywords:** immun checkpoint inhibitors, immunotherapy adverse effect, cancer-immunotherapy, lung cancer, cancer metastasis, incidence and prevalence, drug induced pneumonitis, non-small cell lung carcinoma (nsclc), non small cell lung cancer, checkpoint inhibitor pneumonitis

## Abstract

Non-small cell lung cancer (NSCLC) is the most common type of lung cancer, often diagnosed at the advanced stage (metastatic). Treatment options for metastatic NSCLC include radiotherapy, chemotherapy, target drug therapy, and immunotherapy. Immunotherapy (utilization of checkpoint inhibitors) boosts the immune system to recognize and destroy cancer cells. However, it is often associated with immune-related complications such as pneumonitis. This review aims to determine the incidence of pneumonitis in metastatic NSCLC patients treated with different immunotherapy drugs. PubMed, Cochrane Library, and Embase databases were scoured for randomized controlled trials (RCTs) until October 2023. Published RCTs with similar research objectives were included, while non-English articles, reviews, case reports, ongoing trials, non-randomized studies, conference abstracts, and studies on small cell lung cancer (SCLC) were excluded. The Cochrane risk-of-bias tool for randomized trials (RoB 2) was used to assess the risk of bias among the included studies. The statistical analyses were performed with the Comprehensive Meta-Analysis software. The subgroup analysis of the 16 included RCTs showed that metastatic NSCLC patients treated with nivolumab and pembrolizumab had a higher incidence of any grade pneumonitis than those treated with atezolizumab (4.5% and 5.1% vs. 1.6%, respectively). Similarly, the incidence of grade ≥3 pneumonitis was higher among patients receiving nivolumab (1.3%) and pembrolizumab (2.4%) than those receiving atezolizumab (0.7%). Furthermore, the subgroup analysis showed that patients with naive-treated NSCLC on immunotherapy had a higher incidence of any grade pneumonitis than those with previously treated NSCLC (6.5% vs. 3.9%). Treatment-naive patients recorded higher grade ≥3 pneumonitis incidences than those previously treated (3.1% vs. 1.3%). Programmed death 1 (PD-1) inhibitors (i.e., pembrolizumab and nivolumab) have higher incidences of pneumonitis than programmed death-ligand 1 inhibitors (atezolizumab).

## Introduction and background

Lung cancer is among the deadliest malignancies worldwide and is the leading contributor to cancer-related deaths among women and men [1]. Non-small cell lung cancer (NSCLC), a subdivision of lung cancer, equals the majority of incidences, with surgery being the leading management for fully developed NSCLC [2]. It is the second leading cancer type in the USA in terms of incidence and the leading in terms of attributable deaths [3]. Study evidence indicates that fully developed NSCLC has a poor prognosis with low one- and five-year survival rates [4]. Despite the utilization of paclitaxel- and platinum-based chemotherapeutic agents and recent addition of biologics, such as vascular endothelial growth factor inhibitors (ramucirumab and bevacizumab), there are still poor survival outcomes [5]. Lately, the utilization of immunotherapy-based intervention regimens has indicated better outcomes in enhancing progression-free and overall survival in NSCLC.

With the development and evolution in the treatment and management of NSCLC in recent decades, the approval of targeted therapies, including immune checkpoint inhibitors (ICIs) and tyrosine kinase inhibitors (TKIs), has led to promising long-term survival, reducing the role of traditional chemotherapy [3,6,7]. Despite their promising survival outcomes, these therapies are linked to possibly fatal treatment-induced adverse events, such as pneumonitis. Immunotherapy-induced pneumonitis was characterized by the emergence of unusual imaging results, with or without respiratory symptoms, after starting immunotherapy and is classified according to the Common Terminology Criteria for Adverse Events (CTCAE) [7].

Adverse events, including interstitial lung abnormalities in treatment-naïve advanced NSCLC patients, are associated with smoking, a well-established risk factor leading to a shorter survival rate among lung cancer patients [8]. Immunotherapy causes life-threatening adverse events, such as pneumonitis, complicating its use among lung cancer patients [9]. Pneumonitis, also referred to as checkpoint inhibitor pneumonitis (CIP), occurs if the foreign irritant substances inflame the minute air sacs in an individual's alveoli (lungs) [10].

The severity of pneumonitis symptoms ranges from mild to life-threatening depending on its grade. According to the National Cancer Institute of the National Institutes of Health, pneumonitis can be asymptomatic (grade 1), symptomatic (grade 2), severe symptoms (grade 3), life-threatening compromise (grade 4), or death related to adverse outcomes (grade 5) [11,12]. Research evidence shows it is a problematic condition among immune-related adverse events (irAEs) [5,11].

CIP mechanisms remain indistinguishable; however, they are believed to be associated with immune dysregulation caused by ICIs [12]. Higgins and Thompson [13] highlighted four possible mechanisms underlying CIP as an irAE. Foremost, adverse events can be associated with escalated T-cell activity against cross-antigens in normal and tumor tissues. Increased activated alveolar T-cell percentage and attenuation of the anti-inflammatory Treg phenotype might contribute to T-cell activity dysregulation, triggering irAE [14]. Second, increased preexisting autoantibody levels might contribute to irAEs. Fehrenbacher et al. indicated that preexisting antinuclear antibodies, anti-rheumatoid factor antibodies, anti-thyroid peroxidase antibodies, and anti-thyroglobulin are possibly associated with the advancement of irAEs in NSCLC patients [15]. Third, increased inflammatory cytokine levels are related to the appearance of irAEs. A patient diagnosed with NSCLC with advanced CIP after atezolizumab treatment is noted to have escalated interleukin-6 (IL-6) and C-reactive protein levels compared to baseline levels [16]. Similarly, cytokines can be biomarkers for adverse measures, and their escalated expression is related to acute ICI toxicity [17,18]. Another possible mechanism is the direct binding of anti-CTLA-4 (cytotoxic T lymphocyte-associated antigen) antibodies with CTLA-4 on normal tissues, for instance, the pituitary gland. This method might also explain why pituitary inflammation is a specific adverse event of anti-CTLA-4 antibody [19].

Pneumonitis has been recognized among patients treated with programmed death ligand 1 (PD-L1)/programmed death 1 (PD-1) ICIs [7]. Although most patients respond to oral corticosteroids, some might develop substantial dyspnea and hypoxia, necessitating NSCLC therapy discontinuation, supplemental oxygen, or intravenous corticosteroids and additional immunosuppressive interventions, such as mycophenolate mofetil, cyclophosphamide, or infliximab [7]. Even though it barely occurs, high-grade (grade 3/4) pneumonitis is linked to significant morbidity and mortality in a small proportion (1.0%) of the affected patients [8].

Pneumonitis incidence has been documented to be between 3.0% and 5.0% in clinical trial evaluations [8,9], but pneumonitis incidence in NSCLC is not yet known, especially across patients treated with different immunotherapy drugs. Only a few meta-analyses and systematic reviews have been performed to evaluate the safety and efficacy of palliative radiotherapy (pRT) and ICIs in metastatic NSCLC patients [10] and risk of pneumonitis among patients receiving durvalumab treatment and radiotherapy [11]. Consequently, there is a need to evaluate the available evidence regarding the differences in incidences among NSCLC patients treated with various immunotherapy drugs. This systematic review and meta-analysis was conducted to understand the difference in pneumonitis incidences in metastatic NSCLC patients treated with different immunotherapy drugs.

## Review

Methods

Study Design

This study followed the Preferred Reporting Items for Systematic Reviews and Meta-Analyses (PRISMA) guidelines.

Eligibility Criteria

Predetermined eligibility criteria were developed to assess the search records. Included and excluded studies were based on the following conditions (Table 1).

**Table 1:** Inclusion and exclusion criteria developed to screen the search records. SCLC: small cell lung cancer; NSCLC: non-small cell lung cancer; RCTs: randomized controlled trials.

Inclusion criteria	Exclusion criteria
English-published studies to reduce the errors that may result from translating scientific terms	Studies published in a language other than English
Studies assessing pneumonitis incidences among metastatic NSCLC patients treated with immunotherapy	Studies assessing pneumonitis incidences among SCLC patients
RCTs aiming to assess pneumonitis incidences among NSCLC patients	Case reports, case series
	Secondary studies: systematic reviews, meta-analyses, scoping reviews, and narrative reviews. Systematic reviews and meta-analyses were excluded since they do not have the aggregated data about patients to perform the analysis. Similarly, the meta-analyses and systematic reviews can compare different interventions, making it impossible to plot pooled outcomes in a forest plot.

Information Sources and Search Strategy

The reviewers performed an electronic search to locate relevant studies in PubMed, Embase, and the Cochrane Library. An extensive search was performed on these databases for RCTs published up to November 2023. The following search terms were used in Embase, PubMed, and Cochrane Library to identify relevant articles: ((non-small cell lung cancer) AND (pneumonitis)) AND (immunotherapy). Detailed search terms for the three databases are shown in Appendix 1. There was no date (year) restriction in search terms for all the databases.

Data Extraction

The eligible studies were analyzed, and data were abstracted for reviewing and analysis. The extracted data from the eligible studies were study ID (author(s) and publication year), patient characteristics (sample size (patients with metastatic NSCLC treated with immunotherapy only) and median age), follow-up period, immunotherapy agents utilized, comparator/control intervention, number of patients with pneumonitis of any grade, and number of patients with pneumonitis of grade ≥3.

Risk of Bias Assessment

The risk of bias assessment was performed via the Cochrane RoB 2 tool for RCTs, according to the Cochrane Handbook for Systematic Reviews of Interventions [12]. RoB 2 instrument has five aspects utilized to generate the overall risk of bias: (1) randomization process, (2) deviations from the intruded intervention, (3) missing outcome data, (4) measurement of the outcome, and (5) selection of the reported result. Each aspect was assessed with either of the following options: "high risk of bias," "low risk of bias," or "some concerns." Following each aspect evaluation, studies were categorized with a "low risk of bias" judgment if they had one "some concerns" in five of the domains, "Some concerns" judgment if they had two or more "some concerns" in five of the domains, or a "high risk of bias" judgment if they had one "high risk of bias" in one domain.

Statistical Analysis

The random effects model was used to sum up the effect size in various studies. The effect size was reported regarding event rate with a 95% CI. The heterogeneity in studies was evaluated using the *I*^2^ statistics, with a significance level of <0.05. Substantial heterogeneity was ascertained if the value of *I*^2^ statistics was more than 75%, moderate if the value was between 50% and 75%, and low heterogeneity if less than 50% [13]. All the analyses were performed using the Comprehensive Meta-Analysis (CMA) software version 3 (Englewood, USA).

Results

**Figure 1:**
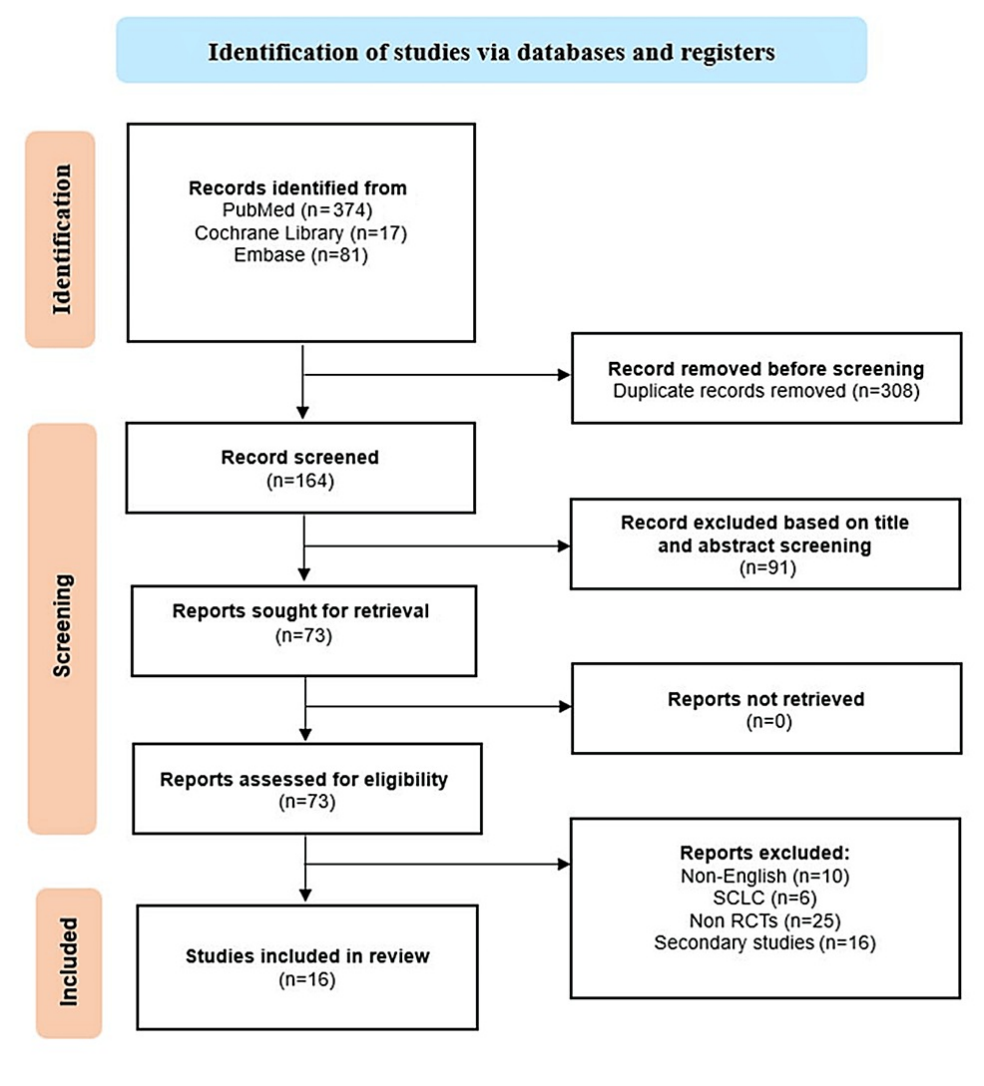
PRISMA flow diagram of search strategy. PRISMA: Preferred Reporting Items for Systematic Reviews and Meta-Analyses.

An initial search of the three electronic databases yielded a total of 474 records. The initial scanning led to the exclusion of 308 articles due to duplication. The remaining 164 articles were screened based on titles and abstracts, leading to the exclusion of 91 articles. The remaining 73 articles were sought for retrieval, and all were retrieved. They were subjected to screening based on the eligibility criteria, and 57 articles were excluded. The reasons for exclusion were non-English (n=10), SCLC (n=6), non-RCTs (n=16), and secondary studies, including systematic reviews, meta-analyses, and narrative reviews (n=25). A PRISMA flow chart of the study selection is shown in Figure 1.

Study Characteristics

The baseline and relevant characteristics of each study that met the inclusion criteria are summarized in Table 2. The 16 studies included in the meta-analysis were conducted between 2015 and 2021, with sample sizes ranging between 52 and 811 patients. Cumulatively, a total sample of 5,662 patients was included in this meta-analysis. The immunotherapy agents evaluated in the studies included nivolumab, atezolizumab, and pembrolizumab.

**Table 2:** Included study characteristics. NSCLC: non-small cell lung cancer; NR: not reported.

Author ID	Sample size (patients with metastatic NSCLC treated with immunotherapy only)	Mean/median age of patients receiving immunotherapy (years)	Follow-up duration (months)	Immunotherapy agent	Comparator treatment	Number of patients with pneumonitis of any grade	Number of patients with pneumonitis of grade ≥3	Type of NSCLC treated with immunotherapy	Outcomes
Felip et al. 2020 [14]	811	66	18	Nivolumab	None	38	5	Previously treated	Nivolumab is active and best tolerated in NSCLC patients with only 0.7% grade ≥3 pneumonitis
Fehrenbacher et al. 2018 [15]	609	63	28	Atezolizumab	Docetaxel	6	4	Previously treated	Fewer patients under atezolizumab (14.9%) experienced grade ≥3 pneumonitis than patients under docetaxel (42.4%)
Wu et al. 2019 [16]	337	60	8.8	Nivolumab	Docetaxel	15	4	Previously treated	Grade ≥3 pneumonitis events were 10.0% with nivolumab and 48.0% with docetaxel
Mok et al. 2019 [17]	636	NR	12.8	Pembrolizumab	Chemotherapy	43	20	Naïve	Grade ≥3 pneumonitis was 8% in pembrolizumab-treated patients and 3% in chemotherapy patients
Borghaei et al. 2021 [18]	418	NR	64.5	Nivolumab	Docetaxel	15	4	Previously treated	Any grade pneumonitis was 3.6% and 1.0% in grade ≥3 among nivolumab patients
Fehrenbacher et al. 2016 [19]	144	62	14.8	Atezolizumab	Docetaxel	4	1	Previously treated	Atezolizumab-related grade≥3 pneumonitis was 2% among patients and 3% in any grade
Herbst et al. 2016 [20]	682	63	13.1	Pembrolizumab	Docetaxel	31	14	Previously treated	Any grade of pembrolizumab-related pneumonitis was 5% and 2% for grade ≥3
Gettinger et al. 2016 [21]	52	67	14.3	Nivolumab	None	3	1	Naïve	Any grade nivolumab monotherapy-related pneumonitis was 6% and 2% in grade ≥3
Garon et al. 2015 [22]	495	NR	10.9	Pembrolizumab	None	18	9	Previously treated	Any grade of pembrolizumab-related pneumonitis was 3.6% and 1.8% for grade ≥3
Reck et al.2016 [23]	154	64.5	11.2	Pembrolizumab	Chemotherapy	9	4	Naïve	5.8% of pembrolizumab patients experienced any grade pneumonitis, and 2.6% grade ≥3. 0.7% of chemotherapy patients experienced any grade pneumonitis, and 0.7% grade ≥3
Rizvi et al.2015 [24]	117	65	8	Nivolumab	None	6	4	Previously treated	Pneumonitis was 5% (any grade) and 3% (grade≥3) among nivolumab-treated patients
Brahmer et al. 2015 [25]	135	62	11	Nivolumab	Docetaxel	6	0	Previously treated	Pneumonitis 5% (any grade) and 0% (grade≥3). Docetaxel had no pneumonitis incidences in any grade and grade ≥3
Borghaei et al. 2015 [26]	292	61	13.2	Nivolumab	Docetaxel	8	3	Previously treated	Any grade pneumonitis was 3% (nivolumab) and <1% (docetaxel)
Gettinger et al. 2015 [27]	129	65	39	Nivolumab	None	10	3	Previously treated	Pneumonitis 3% (grade ≥3)
Hui et al. 2017 [28]	101	68	22.2	Pembrolizumab	None	NR	4	Naïve	Pneumonitis 3.0% (grade ≥3)
Leighl et al. 2019 [29]	550	NR	34.5	Pembrolizumab	None	NR	10	Previously treated and Naïve	Grade ≥3 pneumonitis adverse events was 2% among pembrolizumab-treated patients

*Risk of B* *ias*

**Figure 2:**
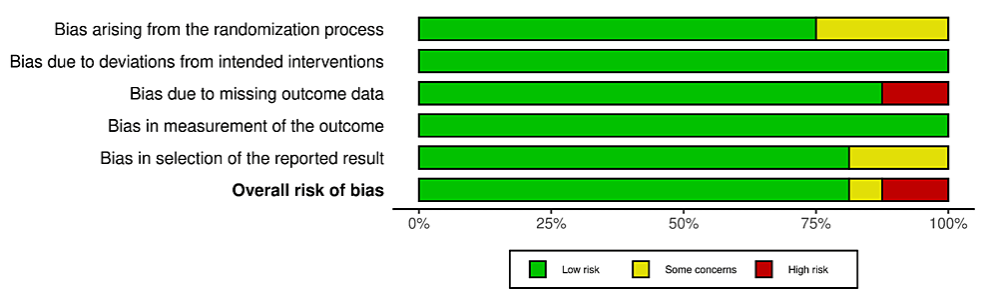
Summary of the risk of bias for each domain among the included studies.

**Figure 3:**
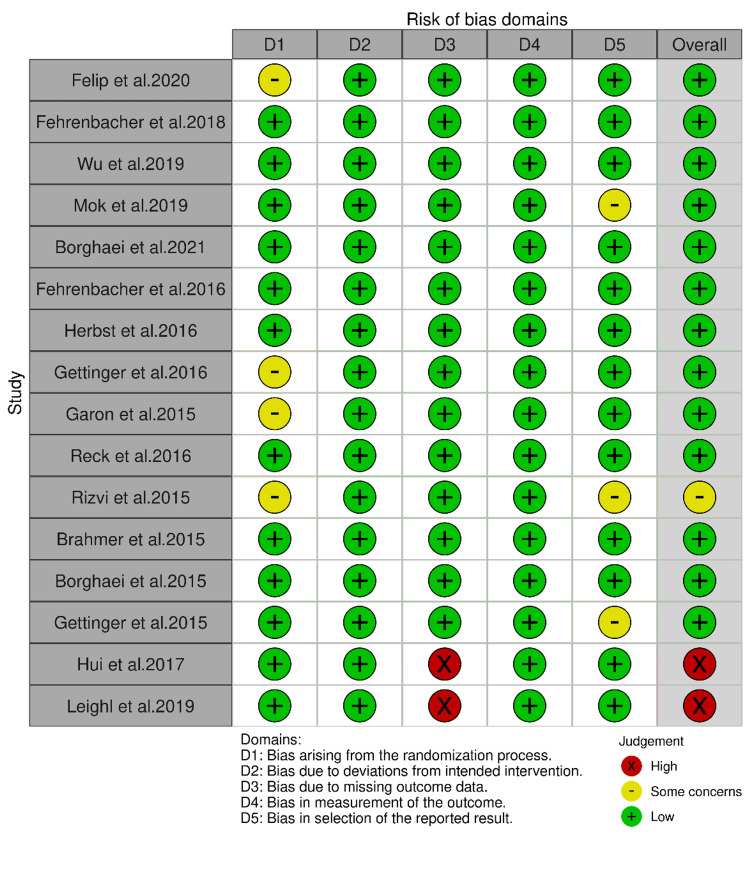
Risk of bias for each study via the revised RoB tool for RCTs. RCTs: randomized controlled trials. Felip et al. 2020 [14], Fehrenbacher et al. 2018 [15], Wu et al. 2019 [16], Mok et al. 2019 [17], Borghaei et al. 2021 [18], Fehrenbacher et al. 2016 [19], Herbst et al. 2016 [20], Gettinger et al. 2016 [21], Garon et al. 2015 [22], Reck et al. 2016 [23], Rizvi et al. 2015 [24], Brahmer et al. 2015 [25], Borghaei et al. 2015 [26], Gettinger et al. 2015 [27], Hui et al. 2017 [28], Leighl et al. 2019 [29].

Figures 2 and 3 show the risk of bias summary for each domain and for each study, respectively. Thirteen studies showed a low risk of bias [14-23,25-27], two showed high risk of bias [28,29], and one study showed some concerns [24].

Immunotherapy Drugs Treatment

**Figure 4:**
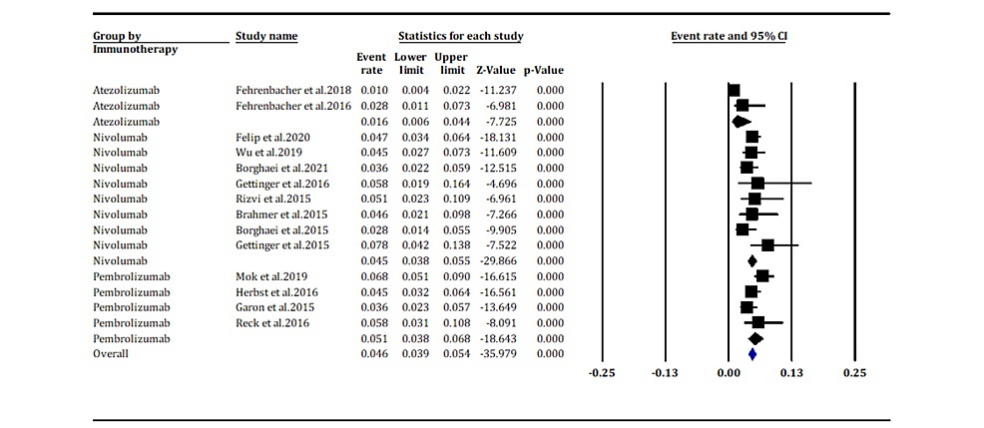
Pneumonitis incidence rate of any grade according to the immunotherapy drug. Fehrenbacher et al. 2018 [15], Fehrenbacher et al. 2016 [19], Felip et al. 2020 [14], Wu et al. 2019 [16], Borghaei et al. 2021 [18], Gettinger et al. 2016 [21], Rizvi et al. 2015 [24], Brahmer et al. 2015 [25], Borghaei et al. 2015 [26],  Mok et al. 2019 [17], Herbst et al. 2016 [20], Garon et al. 2015 [22], Reck et al. 2016 [23].

Pneumonitis incidences based on immunotherapy drugs (any grade): Fourteen studies assessed the incidence rate of any grade pneumonitis among patients based on immunotherapy drugs. The subgroup analysis indicates a higher incidence of any grade pneumonitis in patients treated with PD-1 inhibitors, i.e., pembrolizumab and nivolumab: incidence rate of 5.1% (95% CI [3.8%, 6.8%]) and 4.5% (95% CI [3.8%, 5.5%]), respectively, than PD-L1 inhibitor, i.e., atezolizumab: an event rate of 1.6% (95% CI [0.6%, 4.4%]) (Figure 4). The difference in incidence rates between the groups was statistically significant (p < 0.05). Overall, the heterogeneity among the studies was moderate (*I*^2^ = 57.008%; p = 0.004).

**Figure 5:**
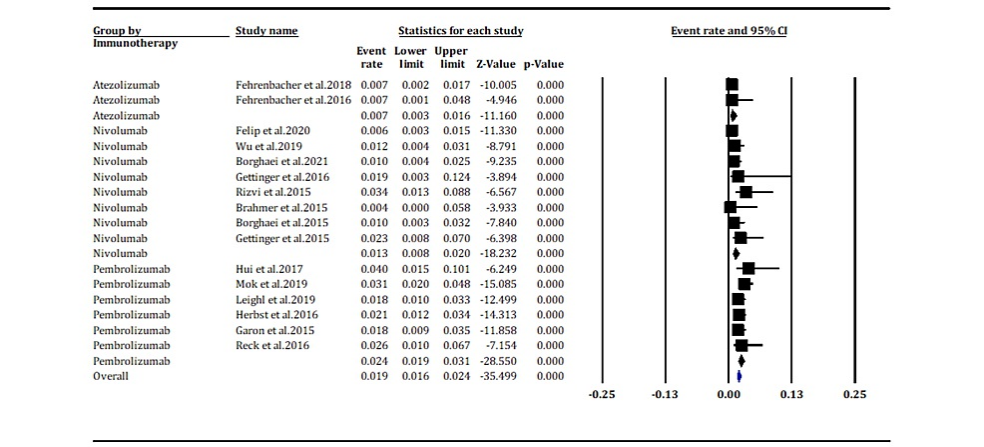
Incidence of grade ≥3 pneumonitis according to immunotherapy drug (overall p-value; p < 0.05). Fehrenbacher et al. 2018 [15], Fehrenbacher et al. 2016 [19], Felip et al. 2020 [14], Wu et al. [16], Borghaei et al. 2021 [18], Gettinger et al. 2016 [21], Rizvi et al. 2015 [24], Brahmer et al. 2015 [25], Borghaei et al. 2015 [26], Gettinger et al. 2015 [27], Hui et al. 2017 [28], Mok et al. 2019 [17], Leighl et al. 2019 [29], Herbst et al. 2016 [20], Garon et al. 2015 [22], Reck et al. 2016 [23].

Pneumonitis incidences based on immunotherapy drugs (grade ≥3): Sixteen studies reported grade ≥3 pneumonitis incidence among patients based on the immunotherapy drugs. The pooled subgroup analysis indicates a higher incidence of grade ≥3 pneumonitis in patients treated with PD-1 inhibitors, i.e., pembrolizumab and nivolumab: event rate of 2.4% (95% CI [1.9%, 3.1%]) and 1.3% (95% CI [0.8%, 2.0%]), respectively, than PD-L1 inhibitor, atezolizumab: event rate of 0.7% (95% CI [0.3%, 1.6%]) (Figure 5). The difference in incidence rates between the immunotherapy drugs was statistically significant (p < 0.05). The results displayed low heterogeneity (*I*^2^ = 43.817%; p = 0.031).

NSCLC Treatment

**Figure 6:**
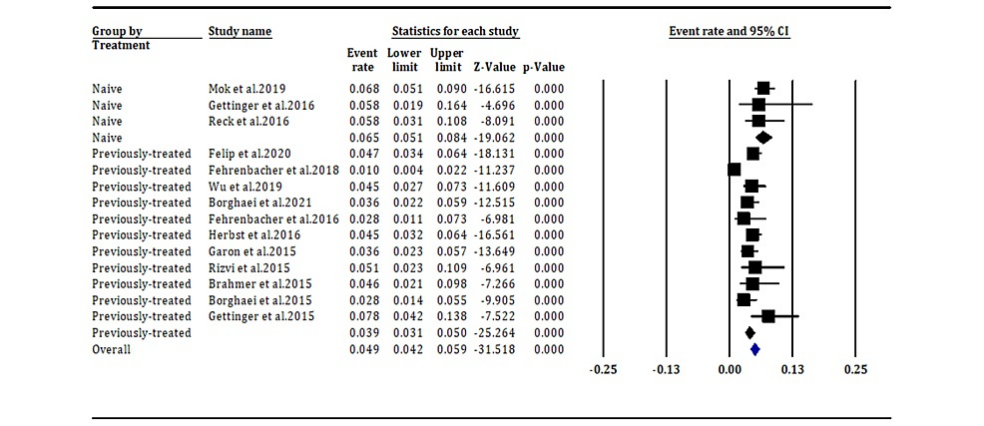
Incidence of pneumonitis of any grade according to the NSCLC treatment (overall p-value, p < 0.05). Mok et al. 2019 [17], Gettinger et al. 2016 [21], Reck et al. 2016 [23], Felip et al. 2020 [14], Fehrenbacher et al. 2018 [15], Wu et al. 2019 [16], Borghaei et al. 2021 [18], Fehrenbacher et al. 2016 [19], Herbst et al. 2016 [20], Garon et al. 2015 [22], Rizvi et al. 2015 [24], Brahmer et al. 2015 [25], Borghaei et al. 2015 [26], Gettinger et al. 2015 [27].

Pneumonitis incidence according to NSCLC treatment (any grade): The subgroup analysis indicates that patients with treatment-naive metastatic NSCLC on immunotherapy have higher incidences of any grade pneumonitis, with an event rate of 6.5% (95% CI [5.1%, 8.4%]), than those with previously treated metastatic NSCLC, with an event rate of 3.9% (95% CI [3.1%, 5.0%]) (Figure 6).

**Figure 7:**
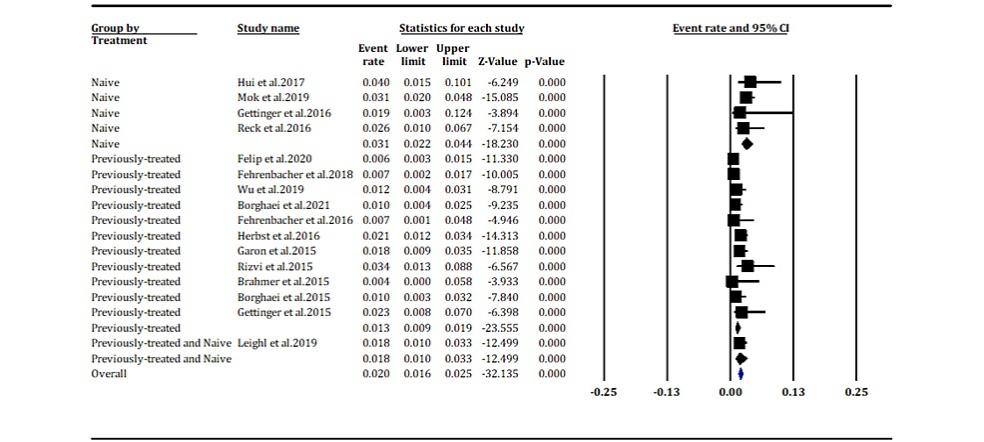
Pneumonitis incidence according to NSCLC treatment (grade ≥3) (overall p-value, p < 0.05). Hui et al. 2017 [28], Mok et al. 2019 [17], Gettinger et al. 2016 [21], Reck et al. 2016 [23], Felip et al. 2020 [14], Fehrenbacher et al. 2018 [15], Wu et al. 2019 [16], Borghaei et al. 2021 [18], Fehrenbacher et al. 2016 [19], Herbst et al. 2016 [20], Garon et al. 2015 [22], Rizvi et al. 2015 [24], Brahmer et al. 2015 [25], Borghaei et al. 2015 [26], Gettinger et al. 2015 [27].

Pneumonitis incidence according to NSCLC treatment (grade ≥3): >The subgroup analysis indicates that patients with treatment-naive metastatic NSCLC on immunotherapy have higher incidences of grade ≥3 pneumonitis, with an event rate of 3.1% (95% CI [2.2%, 4.4%]), than those with previously treated metastatic NSCLC, with an event rate of 1.3% (95% CI [0.9%, 1.9%]) (Figure 7).

Discussion

This study aimed to assess the incidence rate of pneumonitis among metastatic NSCLC patients treated with various immunotherapy drugs. The analyses show that PD-1 inhibitors (pembrolizumab and nivolumab) have significantly higher incidences of pneumonitis than PD-L1 inhibitors (atezolizumab). Similarly, pneumonitis incidences among patients on immunotherapy were significantly higher for patients with naive-treated metastatic NSCLC.

Checkpoint inhibitor immunotherapy is gaining popularity and utilization in cancer care [30]. Pneumonitis is among the adverse events in utilizing NSCLC immunotherapy drugs that determine their safety. Such an adverse event can be unpredictable in relation to severity and timing, resulting in significant mortality (occasionally) and morbidity.

Therefore, early detection and understanding of pneumonitis incidence rates can be vital for clinicians and medical practitioners to reduce irreversible lung damage among patients caused by such adverse events [31]. Additionally, evidence has indicated that physicians' knowledge regarding pneumonitis incidence rates among NSCLC patients can assist in devising mechanisms for reducing pneumonitis' impact, improving treatment adherence, and enhancing outpatient outcomes [31]. 

Various studies, including systematic reviews, have evaluated the incidences of several serious adverse events without solely focusing on pneumonitis [8,31,32]. Zhou et al.’s [32] meta-analysis showed that incidences of serious adverse event (rash, hepatobiliary disorders, pneumonitis, and colitis) were 37% in the chemotherapy arm, 37% in PD-L1, and 33% in the PD-1 arm, while 47% in combined groups of PD-L1 plus chemotherapy and 43% in the PD-L1 plus chemotherapy arm [32]. These results are similar to the outcomes of the present analysis, where PD-1 inhibitors had significantly higher pneumonitis incidences than PD-L1 inhibitors. Despite PD-1 inhibitors’ high pneumonitis incidence rate, evidence has shown that pembrolizumab significantly prolongs overall and progression-free survival with less high-grade toxic adverse events in advanced melanoma patients compared to ipilimumab standard care [33]. Also, nivolumab monotherapy offers better patient progress and clinical safety mechanisms (meaningful pneumonitis incidences) in advanced refractory squamous NSCLC [24]. Reck et al. also noted that in comparison with chemotherapy treatment, pembrolizumab experienced more immune-related adverse events (pneumonitis) but had a better overall safety profile [23].

Shankar and Naidoo reported similar findings, showing that patients with PD-L1 monotherapy had a lower incidence of pneumonitis compared to those treated with PD-1 monotherapy [34]. Atezolizumab offers better survival benefits in previously treated NSCLC patients [19]. Its expression on tumor-infiltrating immune and tumor cells is predictive of such an advantage. For an extended follow-up period, atezolizumab shows a favorable safety profile than docetaxel as compared to PD-1 inhibitors [19]. The nature and incidence of treatment-related adverse events of any grade and grade 3 were low even after longer exposure to treatment [19]. Lower pneumonitis incidence among patients treated with PD-L1 may be due to the action mechanism of an anti-PD-1 agent blocking the PD-L1 and PD-L2 interactions, while anti-PD-L1 agents allow interactions between PD-L2 and PD-1 [35].

With any presentation, immune-related adverse events should be contemplated in multiple diagnoses and necessitate emphasis from the clinicians. Pneumonitis is a toxicity of specific concern for doctors managing NSCLC, with a higher incidence of pneumonitis induced by immunotherapy. Risk factors that contribute to higher incidences of pneumonitis may include patients with a smoking history or underlying lung pathology who can develop grade 3 pneumonitis [30]. Similarly, pneumonitis can occur more frequently in patients with renal cell carcinoma and NSCLC than in metastatic melanoma patients [36]. Wu et al. [37] noted that the lowest pneumonitis rates are displayed in patients treated for advanced melanoma (0.720%) and the highest in advanced NSCLC-treated patients (4.70%) [37]. Studies in this meta-analysis and previous research have also shown that incidence varies with various tumor types, with pembrolizumab-induced pneumonitis being less frequent in melanoma (1%) than in NSCLC patients (5%) [20,33,36]. The results of this meta-analysis align with this study's outcomes that pneumonitis incidences among patients on immunotherapy were significantly higher for patients with naive-treated metastatic NSCLC. NSCLC patients are more likely to have lung diseases, for instance, chronic obstructive pulmonary disease, likely increasing their vulnerability to developing higher-grade pneumonitis [38].

Furthermore, pneumonitis incidences are prominent among NSCLC patients, and it can be attributed to poor pulmonary functioning with the burden of tumors among such patients [36]. Patients with PD-L1 and PD-1 inhibitor-induced pneumonitis show lymphocyte infiltration in lung biopsies and predominant lymphocytes in bronchoalveolar lavage [39]. Since pneumonitis is reported as the most common contributor to fatal immune-related adverse events in both PD-L1 and PD-1 inhibitors [40], medical practitioners should enhance pneumonitis management in NSCLC. For instance, healthcare practitioners should decide the treatment duration and the utilization of immune-related drugs based on the severity of the adverse event. There should also be consideration in the re-introduction of ICIs after discontinuation and emphasis on the treatment and diagnosis of each patient [36,41].

Limitations of the Study

Some limitations should be noted when interpreting the results of this study. There is no consensus about the diagnostic criteria for pneumonitis. Hence, determining pneumonitis was based on clinicians’ experience, which can contribute to bias. Two studies by Hui et al. [28] and Leigh et al. [29] had a high risk of bias due to missing outcome data, which were not reported.

## Conclusions

This study has provided specific safety insights into the presentation and incidence of pneumonitis in metastatic NSCLC patients treated with different immunotherapy drugs. The meta-analysis illustrates that PD-1 inhibitors, pembrolizumab and nivolumab, have higher incidences of pneumonitis than PD-L1 inhibitors (atezolizumab). Incidences of pneumonitis among patients on immunotherapy were higher for patients with naive-treated metastatic NSCLC. The reporting of these findings might be essential for clinical decisions, including advising on risk factors like smoking habits, among metastatic NSCLC patients. Early detection of pneumonitis is crucial as it allows for timely intervention, which can prevent further morbidity or mortality. Early diagnosis and management of pneumonitis can significantly reduce the severity of symptoms, improve patient outcomes, and potentially prolong survival. By identifying high-risk patients and implementing preventive measures, healthcare providers can minimize the incidence and impact of pneumonitis, ensuring better overall treatment efficacy and enhancing patients' quality of life​. 

## Appendices

PubMed search terms

("carcinoma, non small cell lung"[MeSH Terms] OR ("carcinoma"[All Fields] AND "non small cell"[All Fields] AND "lung"[All Fields]) OR "non-small-cell lung carcinoma"[All Fields] OR ("non"[All Fields] AND "small"[All Fields] AND "cell"[All Fields] AND "lung"[All Fields] AND "cancer"[All Fields]) OR "non small cell lung cancer"[All Fields] OR (("metastatically"[All Fields] OR "metastatics"[All Fields] OR "metastatization"[All Fields] OR "metastatize"[All Fields] OR "metastatized"[All Fields] OR "metastatizing"[All Fields] OR "secondary"[MeSH Subheading] OR "secondary"[All Fields] OR "metastatic"[All Fields]) AND ("carcinoma, non small cell lung"[MeSH Terms] OR ("carcinoma"[All Fields] AND "non small cell"[All Fields] AND "lung"[All Fields]) OR "non-small-cell lung carcinoma"[All Fields] OR ("nonsmall"[All Fields] AND "cell"[All Fields] AND "lung"[All Fields] AND "cancer"[All Fields]) OR "nonsmall cell lung cancer"[All Fields]))) AND ("pneumonia"[MeSH Terms] OR "pneumonia"[All Fields] OR "pneumonitis"[All Fields]) AND ("immunotherapy"[MeSH Terms] OR "immunotherapy"[All Fields] OR "immunotherapies"[All Fields] OR "immunotherapy s"[All Fields])

Cochrane Library

(metastatic non-small cell lung cancer in Title Abstract Keyword AND pneumonitis in Title Abstract Keyword AND immunotherapy in Title Abstract Keyword)

Embase

(“NSCLC” or “carcinoma” or “non-small cell lung cancer”) AND (pneumonitis) AND (immunotherapy)
